# Episodic memory decline in Parkinson’ s disease: relation with white matter hyperintense lesions and influence of quantification method

**DOI:** 10.1007/s11682-018-9909-x

**Published:** 2018-06-13

**Authors:** Vincent Dunet, Mario Joao Fartaria, Jeremy Deverdun, Emmanuelle Le Bars, Florence Maury, Giovanni Castelnovo, Tobias Kober, Meritxell Bach Cuadra, Christian Geny, Benedicte Marechal, Nicolas Menjot de Champfleur

**Affiliations:** 1 Department of Diagnostic and Interventional Radiology, Lausanne University Hospital, Rue du Bugnon 46, CH-1011 Lausanne, Switzerland; 2 Advanced Clinical Imaging Technology, Siemens Healthcare HC CEMEA SUI DI PI, Lausanne, Switzerland; 3 Signal Processing Laboratory (LTS 5), Ecole Polytechnique Fédérale de Lausanne (EPFL), Lausanne, Switzerland; 4 Department of Neuroradiology, Montpellier University Hospital Center, Montpellier, France; 5 I2FH, Montpellier University Hospital Center, Montpellier, France; 6 Laboratoire Charles Coulomb, CNRS UMR 5221, University of Montpellier, Montpellier, France; 7 Department of Neurology, Montpellier University Hospital Center, Montpellier, France; 8 Department of Neurology, Caremeau University Hospital Center, Nimes, France; 9 Medical Image Analysis Laboratory (MIAL), Centre d’Imagerie BioMédicale (CIBM), Lausanne, Switzerland; 10 Department of Radiology, Caremeau University Hospital Center, Nimes, France

**Keywords:** Episodic memory, Parkinson’s disease, White matter hyperintense lesions, MRI, Quantification

## Abstract

The relation of white matter hyperintense lesions to episodic memory impairment in patients with Parkinson’s disease (PD) is still controversial. We aimed at evaluating the relation between white matter hyperintense lesions and episodic memory decline in patients with PD. In this multicentric prospective study, twenty-one normal controls, 15 PD patients without mild cognitive impairment (MCI) and 13 PD patients with MCI were selected to conduct a clinico-radiological correlation analysis. Performance during episodic memory testing, age-related white matter changes score, total manual and automated white matter hyperintense lesions volume and lobar white matter hyperintense lesions volumes were compared between groups using the Kruskal-Wallis and Wilcoxon signed-rank tests, and correlations were assessed using the Spearman test. MCI PD patients had impaired free recall. They also had higher total, left prefrontal and left temporal white matter hyperintense lesions volumes than normal controls. Free recall performance was negatively correlated with the total white matter hyperintense lesions volume, either manually or automatically delineated, but not with the age-related white matter changes score. Using automated segmentation, both the left prefrontal and temporal white matter hyperintense lesions volumes were negatively correlated with the free recall performance. Early episodic memory impairment in MCI PD patients may be related to white matter hyperintense lesions, mainly in the prefrontal and temporal lobes. This relation is influenced by the method used for white matter hyperintense lesions quantification. Automated volumetry allows for detecting those changes.

## Introduction

Cognitive dysfunction in elderly subjects may be linked to white matter hyperintense lesions (WMHL) related to cerebral small vessel disease (CSVD) (Zhou et al. 2015; Maillard et al. 2012; Smith et al. 2011). In elderly hypertensive patients with subjective memory complaints, higher WMHL volume has been correlated with lower grey matter metabolism, independently of age, gender or grey matter atrophy (Verger et al. 2016). Higher WMHL load has also been reported in mild cognitive impaired (MCI) and demented patients with Parkinson’s disease (PD) (Beyer et al. 2006; Kandiah et al. 2013; Lee et al. 2010; Mak et al. 2015; Slawek et al. 2013; Sunwoo et al. 2014). Episodic memory (EM) is early impaired in PD and may be a risk factor for future cognitive decline (Broeders et al. 2013). The impact of CSVD on EM is however still inconclusive, notably in old-age onset PD patients. A recent critical review of the pertinent literature (Vesely and Rektor 2016) suggested that the controversial results on the contribution of WMHL to cognitive decline in PD might be due to differences in methods of cognitive testing and WMHL load assessment from MRI.

WMHL load can be evaluated either by visual rating or quantitative methods. While visual rating and WMHL volume have been well correlated (Kapeller et al. 2003) and present good interrater reliability (Olsson et al. 2013), an automatized volumetric method for measuring WMHL load is more suitable for evaluating progression of the disease (Prins et al. 2004). WMHL volume on fluid-attenuated inversion recovery (FLAIR) images may be delineated manually, using semi-automated (Dalaker et al. 2009) or fully-automated software (Fartaria et al. 2016; Gibson et al. 2010), this latter demonstrating high accuracy. We thus hypothesized in the present study that early EM decline in elderly MCI patients with newly diagnosed PD could be due to CSVD and that this relation could be influenced by the employed WMHL quantification method.

The aim of the study was to evaluate the relation between WMHL load and EM testing in patients older than 70 years with newly diagnosed PD as well as the impact of the WMHL evaluation method on this interrelation.

## Materials and Methods

### Study population

From December 2011 to June 2016, 27 healthy controls (mean age: 77.6 ± 3.2 years; male: 20) and 67 patients (mean age: 79.6 ± 4.7 years; male: 35) were prospectively enrolled in this multicentric study. Inclusion criteria for patients were: age between 70 and 90 years, symptoms of parkinsonism starting after the age of 70 years, no history of cardiovascular disease. Inclusion criteria for controls were: age between 70 and 90 years, no parkinsonian symptoms, no history of cardiovascular disease. Exclusion criteria were identical for all subjects (controls and patients) and included: previous history of head injury, stroke, neuroinflammatory disorders, myocardial infarct, intra-cranial bleeding, exposure to neuroleptic drugs, psychiatric comorbidity, contraindications to MRI. In order to rule out stroke, intra-cranial bleeding or neuroinflammatory disorders related sequelae, brain MRI or CT examinations available on the institutional image archiving system before inclusion as well as MRI scans performed during the study were reviewed by a neuroradiologist who was not involved in the MRI data processing. All participants underwent brain MRI, a neurological examination to determine the parkinsonism’s subtype and a neuropsychological assessment including EM testing within the same day. The mean delay between patients’ inclusion and clinico-radiological evaluation was 9.4 ± 42.4 days.

### Ethical approval

This study was approved by the Institutional Review Board and conformed with the World Medical Association Declaration of Helsinki. The experiments were undertaken with understanding and written informed consent of each subject.

### Clinical assessment

All participants underwent a neurological and neuropsychological evaluation on the day of brain MRI. Diagnoses were established by a neurologist experienced in parkinsonian syndromes, blinded to the MRI results and according to the following established guidelines: the UK Parkinson’s Disease Society Brain Bank criteria for idiopathic PD, the National Institute of Neurological Disorders and Stroke and the Society for Progressive Supranuclear Palsy (PSP) criteria for PSP, Zijlmans’s criteria along with multiple cardiovascular risk factors and minimal or absent Levodopa response for vascular parkinsonism (VP), the Gilman’s criteria for multiple system atrophy (MSA), and the Lang criteria for corticobasal dementia (CBD) as previously reported (Dunet et al. 2016).

Neuropsychological assessment included testing of five cognitive domains: attention and working memory, executive functions, language, visuospatial abilities and episodic memory. Global cognitive efficiency was assessed using the Mini Mental State Examination (MMSE) score (range 0–30) and the Mattis Dementia Rating Scale (range 0–144), which involves five subsets (attention, initiation, construction, conceptualization, and memory). Attention and working memory were assessed with a auditory-verbal forward and backward span task and the Delis-Kaplan Executive Function System (D-KEFS) Trail Making Test evaluating flexibility; executive functions were assessed with the Frontal Assessment Battery, the french version of the Stroop Victoria test evaluating inhibition, the D-KEFS Trail Making Test evaluating flexibility, the Rey complex figure copy test evaluating planification abilities and the clock-drawing test; language was evaluated with the LEXIS and Isaac’s Set tests; visuospatial functions were assessed with the Rey complex figure copy test. Finally, to assess EM, we used the free/cued recall selective reminding test (FCSRT) (Grober et al. 2010). After a training phase that controls verbal attention and semantic encoding, the study includes three trials of recall for 16 items, separated by 20 s of interference, to explore retrieval. Each trial of recall consists of free recall followed by cued recall. A single delayed free/cued recall trial is finally performed 20 min later. Neuropsychological metrics were recorded as z-scores (free recall) or percentiles (cued recall) compared with results of a healthy population normalized for age and gender. MCI patients were diagnosed according to the Level I of the Movement Disorders Task Force guidelines (Litvan et al. 2012) when at least two neuropsychological tests in the five domains were impaired. The Educational Attainment was also recorded.

### Images acquisition and processing

All subjects underwent brain MRI on a 3 Tesla scanner (MAGNETOM Skyra, Siemens Healthcare, Erlangen, Germany) including an axial unenhanced T1-weighted magnetization-prepared rapid 3D gradient-echo (MPRAGE) sequence (repetition time = 1690 ms, echo time = 2.54 ms, inversion time = 922 ms, turbo actor = 208, flip angle = 9°, slice thickness = 1 mm, 176 slices, isotropic voxel size = 1 mm^3^) and sagittal FLAIR sequence (repetition time = 5000 ms, echo time = 384 ms, inversion time = 1800 ms, flip angle = 120°, slice thickness = 0.9 mm, 160 slices, interpolated voxel size = 0.9 × 0.9 × 0.9 mm^3^).

WMHL load was evaluated by visual rating as well as by both manual and automated volume estimation at the patient level. For visual rating, FLAIR images were reviewed by two neuroradiologists in consensus to measure the severity of cerebral age-related white matter changes (ARWMC) by using the four-point scale of the European Task Force: 0 (no lesion), 1 (focal non confluent lesions ≥5 mm), 2 (beginning confluence of lesions) or 3 (diffuse involvement). For WMHL volume estimation, WMHL were manually contoured on FLAIR images by a single neuroradiologist with 8-years’ experience in neuroimaging with the MRIcron software (https://www.nitrc.org/projects/mricron). Volumes were recorded in milliliters (mL) and as percentage of the total intracranial volume. To ensure reproducibility of the measure, delineation was repeated twice for ten subjects randomly chosen, while respecting a delay of 3 months between the two delineations. Mean delineation time was about 20 min per case (1880 min for all participants).

Finally, WMHL volume was also computed using automated prototype software initially designed for Multiple Sclerosis lesion segmentation (Fartaria et al. 2016). The method consists of two main steps: i) pre-processing, where the images are aligned, skull-striped, corrected for bias field and intensity-normalized; and ii) lesion segmentation, performed by a supervised classifier based on k-nearest-neighbor (k-NN) algorithm. Lesion masks of each subject were obtained through a “leave-one-out” cross-validation on the whole cohort of 94 subjects (see section Study population). Lobar WMHL volumes were estimated by summing up voxels labelled as lesion tissue by the automated algorithm over the left and right pre-frontal, frontal, temporal, parietal and occipital masks obtained by atlas propagation (Schmitter et al. 2015). The computation time for the automated segmentation was 10 min per patient (940 min for all participants) on a standard computer. T1-MPRAGE images were also segmented to estimate the lobar (pre-frontal, frontal, temporal, parietal, occipital) grey matter and hippocampus volumes using the MorphoBox software (Schmitter et al. 2015).

### Statistical analysis

All statistics were performed with the Stata 13.1 software (Stata Corp., College Station, TX, USA). Continuous variables are presented as mean ± standard deviation. Statistical analysis was performed in a two-step manner. First, the correlation and concordance of the three methods to quantify WMHL for all participants (*n* = 94) was evaluated by the Lin’s test and Bland-Altman plot with computation of 95% limits-of-agreement (LOA). Second, a clinico-radiological correlation analysis was performed to identify if there was a significant relation between high WMHL load and clinical assessment, i.e. EM testing. For this analysis, three groups were considered: normal controls (NC), PD patients without MCI (non-MCI PD), and PD patients with MCI (MCI PD). Group comparisons were performed using the Kruskal-Wallis and Wilcoxon tests for continuous variables and the Fisher exact test for proportions. The relation between each neuropsychological metric (FCSRT and Mattis score) and total WMHL load (ARWMC score, total manual and automated WMHL volumes) was assessed by the non-parametric Spearman rho correlation coefficient. We also evaluated the relation between the same neuropsychological metrics and lobar (left/right prefrontal, frontal, temporal, parietal, occipital) WMHL and grey matter (lobar and hippocampus) volumes by the non-parametric Spearman correlation coefficient. All correlation analyses were adjusted for age and gender. An uncorrected *p*-value <0.05 was considered significant. For multiple correlation analyses, correction of the significance level was performed using the Benjamini and Hochberg method with a false discovery rate of 0.1 (Green and Diggle 2007). For these multiple correlation analyses, only results remaining significant after correction of the significance level are reported in the text.

## Results

### Study population

Out of 67 patients who underwent MRI, thirty-four patients met the criteria of idiopathic PD (mean age 79.9 ± 5.2yo; male: 20), eight patients those of VP (mean age 78.0 ± 2.0yo; male: 7), seven patients those of PSP (mean age 79.6 ± 6.7yo; male: 5), four patients those of MSA (mean age: 79.5 ± 3.8yo; male: 2), three patients those of CBD (mean age: 77.3 ± 4.0yo; male: 0), one met criteria for Lewy body dementia (age: 76yo; male: 1), and ten could not yet be classified (mean age: 80.9 ± 4.0yo; male: 4).

Characteristics of subjects included in the clinico-radiological correlation analysis are summarized in Table 1. Out of the 27 controls, six with MCI were excluded. Out of the 34 PD patients, six with dementia were excluded. Overall, 21 NC, 15 non-MCI PD patients and 13 MCI PD patients were included. MCI PD patients were older than NC (*p* = 0.0001). There was no statistically significant difference regarding sex ratio, educational level, time-from-onset of PD symptoms or dopaminergic therapy. MCI PD patients had impaired free and cued recall, global memory, initiation and conceptualization compared with NC (Table 2). They did not have impaired verbal attention and semantic encoding. Regarding grey matter, only occipital volume was lower in MCI PD patients than in NC (*p* = 0.014) and non-MCI PD patients (*p* = 0.019).

**Table 1:** Characteristics of subjects included in the clinico-radiological correlation analysis

Characteristics	NC, *n* = 21	Non-MCI PD, *n* = 15	MCI PD, *n* = 13	p-value
Clinical				
Age (years)	76.7 ± 2.3	78.1 ± 6.1	81.8 ± 3.8*	0.0024
Gender (male/female)	15/6	10/5	7/6	0.60
Educational level (A/B/C/D)	4/3/3/11	3/2/2/8	5/1/1/6	0.88
BMI (kg/m^2^)	25.4 ± 3.5	27.4 ± 13.5	23.3 ± 2.1	0.21
Diabetes (no/yes)	20/1	12/3	12/1	0.42
Hypertension (no/yes)	10/11	9/6	8/5	0.72
Hypercholesterolemia (no/yes)	13/8	13/2	9/4	0.27
Smoking (no/yes)	9/12	8/7	9/4	0.32
Disease duration (years)	–	5.7 ± 3.6	6.5 ± 5.1	0.80
Levodopa medication (no/yes)	–	2/13	4/9	0.26
Daily dose (mg)	–	639 ± 361	551 ± 236	0.87
Freezing of gait (on/off)	–	10/5	7/6	0.70
Imaging				
Total intracranial volume (mL)	1538 ± 135	1538 ± 178	1477 ± 142	0.61
ARWMC score (0–3)	1.4 ± 0.9	1.5 ± 0.8	1.9 ± 1.0	0.34
Total WMHL volume (mL)				
FLAIR manual FLAIR automated	9.2 ± 13.5 8.1 ± 9.2	8.2 ± 7.1 8.8 ± 6.0	18.1 ± 17.1 16.0 ± 13.2	0.13 0.15
Lobar WMHL volumes (mL)				
Left prefrontal Right prefrontal Left frontal Right frontal Left temporal Right temporal Left parietal Right parietal Left occipital Right occipital	0.18 ± 0.21 0.16 ± 0.23 1.08 ± 1.77 1.28 ± 1.87 0.02 ± 0.03 0.04 ± 0.07 1.06 ± 1.72 1.23 ± 1.92 0.05 ± 0.05 0.10 ± 0.14	0.21 ± 0.16 0.18 ± 0.19 1.15 ± 0.94 1.14 ± 0.94 0.04 ± 0.04 0.04 ± 0.08 0.98 ± 1.22 0.79 ± 1.19 0.04 ± 0.06 0.07 ± 0.08	0.40 ± 0.47* 0.31 ± 0.33 2.76 ± 2.61 2.61 ± 2.72 0.15 ± 0.26*^†^ 0.14 ± 0.23 2.25 ± 2.31* 2.65 ± 2.86 0.07 ± 0.05 0.09 ± 0.07	0.10 0.16 0.10 0.27 0.0006 0.12 0.09 0.13 0.21 0.30

*p < 0.05 compared with NC. ^†^p < 0.05 compared with non-MCI PD patients

For Educational level, A = End primary school certificate, B = Mid secondary school certificate, C = End secondary school certificate, D = University graduation. *ARWMC* = age-related white matter changes, *BMI* = body mass index, *MCI* = mild cognitive impairment, *NC* = normal controls, *PD* = Parkinson’s disease, *WMHL* = white matter hyperintense lesions

**Table 2:** Neuropsychological tests

Tests (normalized scores)	NC, n = 21	Non-MCI PD, n = 15	MCI PD, n = 13	p-value
FCSRT				
Immediate recall (percentiles)	42.1 ± 24.2	36.5 ± 24.4	29.3 ± 29.1	0.28
Free recall 1 (z-scores)	0.59 ± 1.07	0.13 ± 0.97	−0.28 ± 0.87*	0.065
Cued recall 1 (percentiles)	58.5 ± 25.2	54.9 ± 28.8	36.4 ± 27.1*	0.085
Free recall 2 (z-scores)	1.03 ± 0.68	0.04 ± 0.99	−0.34 ± 0.84*	0.0003
Cued recall 2 (percentiles)	53.6 ± 18.2	47.9 ± 26.2	35.1 ± 26.9*	0.12
Free recall 3 (z-scores)	0.75 ± 0.74	−0.02 ± 1.05	−0.44 ± 1.23*	0.0076
Cued recall 3 (percentiles)	33.0 ± 19.9	35.8 ± 22.8	35.4 ± 30.6	0.93
Delayed free recall (z-scores)	1.38 ± 0.70	0.41 ± 0.79	−0.43 ± 1.71*	0.0004
Delayed cued recall (percentiles)	35.0 ± 20.5	39.6 ± 22.4	29.7 ± 26.4	0.35
Mattis total (percentiles)	76.6 ± 19.0	68.7 ± 31.2	42.3 ± 26.7	0.0041
Attention (percentiles)	74.5 ± 17.6	67.9 ± 28.1	50.0 ± 21.5*^†^	0.0085
Initiation (percentiles)	63.2 ± 28.0	58.4 ± 31.4	37.2 ± 29.3*	0.082
Construction (percentiles)	50.6 ± 17.1	49.4 ± 21.3	50.7 ± 20.0	0.99
Conceptualization (percentiles)	75.9 ± 17.1	76.9 ± 18.3	56.9 ± 20.5*^†^	0.025
Memory (percentiles)	69.5 ± 22.2	57.3 ± 35.4	39.8 ± 30.7*	0.057

*p < 0.05 compared with NC. ^†^p < 0.05 compared with non-MCI PD patients

*FCSRT* = free/cued recall selective reminding test, *MCI* = mild cognitive impairment, *NC* = normal controls, *PD* = Parkinson’s disease

### WMHL load estimation

**Fig. 1:**
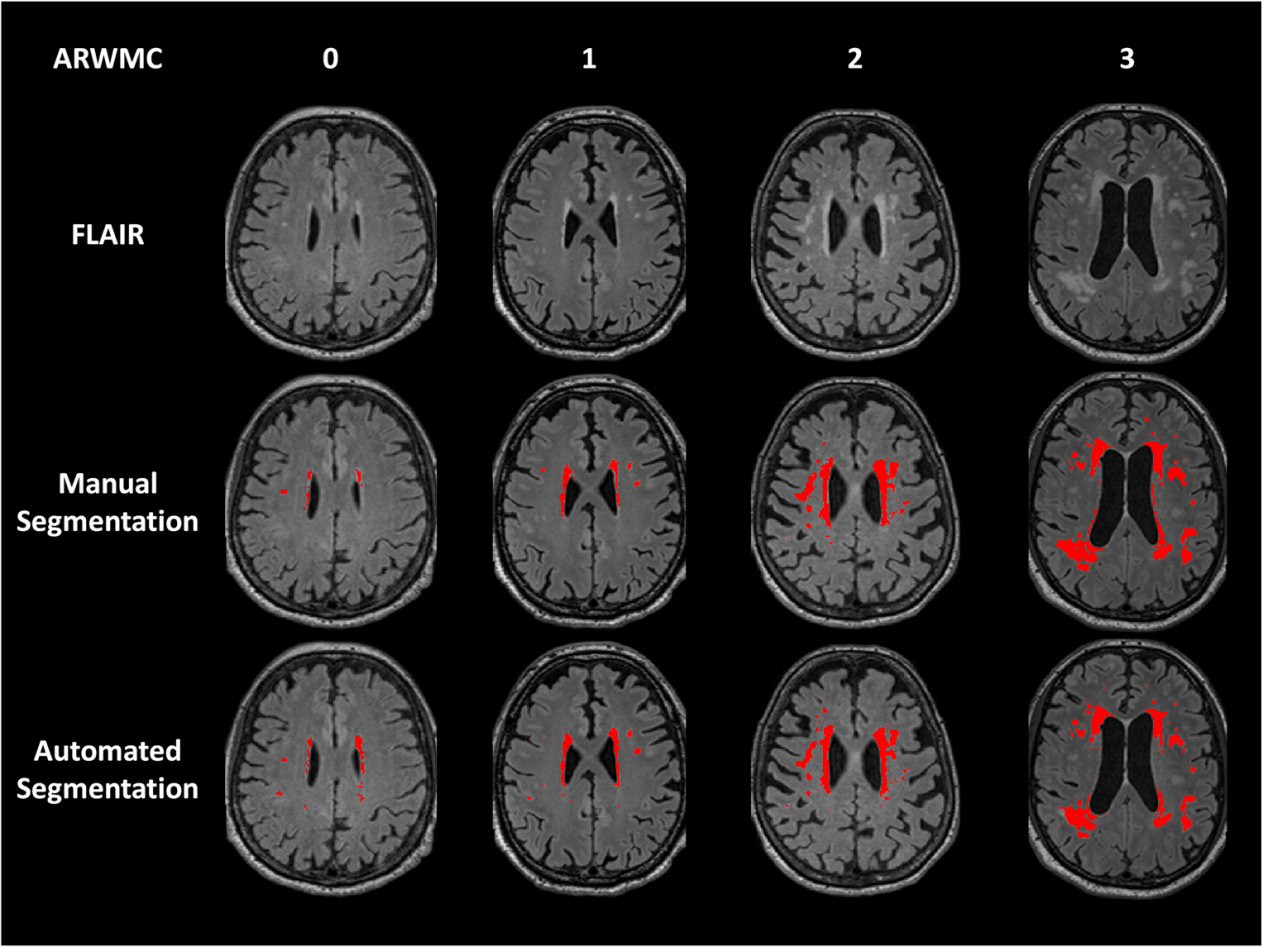
Examples of manual and automated segmentation of white matter hyperintense lesions according to age-related white matter changes score (ARWMC) on FLAIR images

On visual rating, eight subjects had score 0, 34 had score 1, 31 had score 2, and 21 had score 3. Mean total WMHL volumes were 12.7 ± 13.5 mL and 12.5 ± 10.8 mL for manual and automated segmentation, respectively. The concordance between manually and automatically delineated WMHL volumes was excellent (Pearson’s *r* = 0.974, rho_c = 0.950, C_b = 0.975, mean difference = 0.18 ± 3.9 mL, 95%LOA = −7.43–7.79, *p* = 0.65). Manual delineation was highly reproducible (Pearson’s rho = 0.997, rho_c = 0.997, C_b = 0.999, mean difference = 0.07 ± 1.0 mL, 95%LOA = −1.9–2.1, *p* = 0.83). Both were positively correlated with ARWMC score (rho = 0.90, *p* < 0.0001 and rho = 0.87, p < 0.0001 for manual and automated segmentation, respectively, Fig. 1).

ARWMC score was not different between groups while total WMHL volumes tend to be higher in MCI PD patients than in NC (*p* = 0.058 and *p* = 0.063 for manual and automated segmentation, respectively). Total WMHL volume was not correlated with age in our cohort (rho =0.11, *p* = 0.45). Lobar WMHL volumes are displayed in Table 1.

### Clinico-radiological correlation

Detailed results of the correlation analysis between total WMHL volume and neuropsychological metrics are displayed in Table 3. ARWMC score was not correlated with any neuropsychological metric. Total WMHL volume estimated by manual segmentation was correlated with performance during the second cued recall.

**Table 3:** Correlations between total WMHL volume and neuropsychological metrics (n = 49)

Tests	ARWMC score		Manual segmentation		Automated segmentation	
FCSRT	rho	p-value	rho	p-value	rho	p-value
Immediate recall	−0.063	0.67	−0.05	0.76	−0.10	0.49
Free recall 1	0.11	0.45	0.01	0.95	−0.05	0.74
Cued recall 1	−0.05	0.71	−0.09	0.54	−0.15	0.31
Free recall 2	−0.07	0.64	−0.24	0.10	−0.26	0.076
Cued recall 2	−0.18	0.21	−0.41	0.0038*	−0.31	0.034
Free recall 3	−0.16	0.28	−0.29	0.049	−0.33	0.020
Cued recall 3	0.04	0.80	−0.03	0.85	0.04	0.81
Delayed free recall	−0.15	0.32	−0.24	0.11	−0.30	0.047
Delayed cued recall	0.04	0.78	−0.04	0.82	−0.02	0.88
Mattis total	−0.08	0.62	−0.16	0.28	−0.20	0.18
Attention	−0.10	0.50	−0.18	0.22	−0.10	0.49
Initiation	−0.15	0.30	−0.24	0.097	−0.16	0.27
Construction	0.10	0.50	0.08	0.59	0.15	0.32
Conceptualization	0.09	0.55	0.01	0.97	−0.01	0.97
Memory	−0.16	0.28	−0.22	0.15	−0.27	0.07

*ARWMC* = age-related white matter changes, *FCSRT* = free/cued recall selective reminding test, *WMHL* = white matter hyperintense lesions. * indicates significant p-value when correcting for multiple testing with the Benjamini-Hochberg method

Using automated segmentation, performances during the second free recall were correlated with the left prefrontal (rho = −0.37, *p* = 0.009) and left temporal (rho = −0.35, *p* = 0.016) WMHL volumes. Performances during the third free recall were correlated with the left prefrontal (rho = −0.35, *p* = 0.014) and left temporal (rho = −0.39, *p* = 0.0058) WMHL volumes. Delayed free recall performance was correlated with the left prefrontal (rho = −0.37, *p* = 0.013) and left temporal (rho = −0.43, *p* = 0.0035) WMHL volumes. Finally, the left prefrontal WMHL volume was correlated with the Mattis memory subscore (rho = −0.36, p = 0.013). Lobar grey matter and hippocampus volumes were not correlated with any neuropsychological metric (all corrected *p* > 0.15).

## Discussion

In this prospective multicentric study focusing on subjects older than 70 years, PD patients with MCI presented early EM impairment, which was not present in normal controls. EM decline correlated with WMHL volumes, especially in the left prefrontal and temporal lobes, which were accurately quantified by the automated lesion segmentation.

### Relation between WMHL and cognitive decline

WMHL related to CSVD have been correlated with cognitive dysfunction in elderly patients with (Malek et al. 2016) or without PD (Zhou et al. 2015; Maillard et al. 2012; Smith et al. 2011). In a large stroke-free population, WMHL was especially associated with worse memory in subjects older than 70 years, independently of brain atrophy (Dong et al. 2015). Similarly, we found that PD patients older than 70 years with MCI presented early EM decline compared with NC, particularly during free recall testing. MCI PD patients were older than NC but all neuropsychological metrics were normalized for age and gender. Thus, age-related confounds do not account for any of the observed differences in EM performance or for the relation between EM and WMHL. Indeed, total WMHL volume was negatively correlated with EM performance. Our findings thus confirm that WMHL due to CSVD may result in worse EM also in subjects older than 70 years with PD. This is in agreement with two other studies (Kandiah et al. 2013; Lee et al. 2010), which demonstrated that high WMHL volume is associated with low memory performance in PD patients, regardless of age, gender, education status, cardiovascular risk factors, disease duration, or dopaminergic therapy – as in our study. Additionally, we found that EM decline was correlated with WMHL volumes in the left prefrontal and temporal lobes, but not with lobar grey matter or hippocampus volumes, which has never been reported in PD patients. Higher WMHL volumes in the prefrontal lobe may result in lower activity in the prefrontal, temporal and cingulate cortex during EM tasks as demonstrated by Nordahl and co-workers in healthy individuals (Nordahl et al. 2006). As in NC (Lancaster et al. 2016) and in MCI non-PD patients (Remy et al. 2015), temporal white matter microstructure disruption due to regional WMHL may also account for early EM impairment in MCI PD patients. Spatial distribution of WMHL could thus sustain EM decline in MCI PD patients. Moreover, we found that PD patients did not have impaired verbal attention and semantic encoding and that attention was not significantly correlated with total or lobar WMHL volumes. This is in agreement with Dalaker et al. (2009) who found no significant relation between total volume or spatial distribution of WMHL and attention-executive function in PD. Notably, the same authors did not explore the relation between WMHL and EM testing. In three other studies, there was no significant relation between cognitive performance and WMHL severity (Slawek et al. 2013, 2008) or between the progression of WMHL and progression to higher category of cognitive impairment (Gonzalez-Redondo et al. 2012). It is worth mentioning that these studies included PD patients with dementia and/or long disease duration, thus potentially mixing CSVD and PD-related neurodegeneration effects. Indeed, Jones and co-workers (2017) recently highlighted that PD and cardiovascular risk factors are independent risk factors for cognitive impairment. Sunwoo et al. (2014) also found that total WMHL volume is an independent predictor of conversion from MCI to dementia in PD patients. Overall, this suggests that, at the early stage of PD, CSVD rather than PD-related neurodegeneration could induce EM decline. At a later stage, CSVD could still contribute to early conversion to dementia along with PD-related neurodegeneration.

### Impact of WMHL evaluation method

In a recent critical literature review, Vesely and Rektor (2016) suggested that controversial results on the contribution of WMHL to PD cognitive decline might be due to methodological differences for assessing WMHL on MR images. Indeed, in PD patients, WMHL severity has alternatively been evaluated using qualitative (Rodriguez-Oroz et al. 2009; Ng et al. 2012) or semi-quantitative (Beyer et al. 2006; Gonzalez-Redondo et al. 2012; Lee et al. 2010; Slawek et al. 2008) visual rating as well as semi-automated (Dalaker et al. 2009; Mak et al. 2015) or automated (Sunwoo et al. 2014; Kandiah et al. 2013) volumetric estimation. Compared with NC, we did not find higher WMHL load in MCI PD patients using ARWMC visual rating as several previously published studies (Gonzalez-Redondo et al. 2012; Rodriguez-Oroz et al. 2009; Slawek et al. 2008). Using manual or automated segmentation, total WMHL volume tended to be higher in MCI PD patients than in NC, in agreement with the results of Dalaker and co-workers (2009). Two studies (Kandiah et al. 2013; Mak et al. 2015) additionally found higher total WMHL volume in MCI PD patients compared with non-MCI PD patients. Although manual segmentation was highly reproducible, the performance of automated segmentation was excellent with a notable gain in processing time. This provides evidence that automated WMHL segmentation has the potential to accurately measure WMHL volume in PD patients, as it is the case in patients with multiple sclerosis (Fartaria et al. 2016; Gibson et al. 2010). As suggested by Vesely and Rektor (2016), the type of WMHL estimation method influenced the clinico-radiological correlation analysis in our study. On the one hand, we did not find any significant relation between ARWMC score and neuropsychological metrics, as most studies that used simple visual rating (Gonzalez-Redondo et al. 2012; Rodriguez-Oroz et al. 2009). On the other hand, we found a significant relation between EM metrics and total WMHL volume using both manual and automated segmentation. This is in agreement with three studies, including one that used semi-quantitative visual rating (Lee et al. 2010), one that used semi-automated volume quantification (Mak et al. 2015) and one that used automated volume quantification (Kandiah et al. 2013). Interestingly, we first report a correlation between EM decline of PD patients and WMHL volumes in the left prefrontal and temporal lobes, which can only be reliably obtained by an automated segmentation. This overall suggests that automated WMHL volume quantification is fast, reproducible and more suitable to evaluate the relation between EM impairment and CSVD severity.

### Study limitations

This study has several limitations. The sample size in the clinico-radiological correlation analysis was small (*n* = 49) due to the very stringent inclusion and exclusion criteria for the elderly patients’ population. This, however, avoided confounding factors such as stroke, haemorrhage, or traumatic cerebral disease. Our results were moreover independent of age, gender, educational attainment, cardiovascular risk factors, disease duration, and dopamine therapy. Nevertheless larger studies are needed to confirm our results. Regarding the diagnosis of VP, recent recommendations propose three subtypes: post-stroke VP, insidious VP and mixed PD/CVD (Rektor et al. 2018). While post-stroke VP patients were formally excluded from our study we used modified Zijlman’s criteria to diagnose VP patients, which fit criteria for the insidious VP subtype. In the present study, PD patients and mixed PD/CVD patients were enrolled in the same group due to the absence of clear validation of these new criteria and because nuclear medicine imaging was not available in this cohort. As we based our correlation analyses on a WMHL location-free hypothesis, our results are still valuable. Whether automated WMHL volume could help distinguishing between mixed PD/CVD patients and PD patients without CVD should be investigated. Longitudinal data were not recorded and are needed to evaluate the impact of WMHL volume on the time course of EM decline. As discussed above, it was demonstrated that WMHL is an independent predictor of conversion from MCI to dementia in PD patients (Sunwoo et al. 2014). Although Maillard et al. (2012) demonstrated that a 1 mL/year increase in global WMHL volume is associated with an additional 0.7 SD/year of subsequent EM decrease in non-PD subjects, it remains unclear whether WMHL volume follow-up could predict the individual time course of EM decline in PD patients. While effect of cognitive training on memory remained uncertain in a recent meta-analysis (Leung et al. 2015), it is also unknown whether automated WMHL volumetry could be used as a marker to select and follow-up patients who could benefit from cognitive rehabilitation, aggressive cardiovascular risk factors control, or other alternative therapy. This needs further investigations.

## Conclusion

Overall, our results indicate that CSVD may contribute to early EM impairment in MCI PD patients older than 70 years. The relation between neuropsychological metrics and CSVD severity is influenced by the method of assessing WMHL on MRI images. Automated quantification of WMHL volume is reliable and may have diagnostic, prognostic and therapeutic implications in PD patients.
